# The Incidence of Various Antiphospholipid Antibodies, Measured by Commercial-Based Laboratory, with Recurrent Spontenous Abortion and the Impact of Their Profiles on Reproductive Outcome with Active Anticoagulant Therapy

**DOI:** 10.5402/2012/819356

**Published:** 2012-03-12

**Authors:** Nagayoshi Umehara, Tadao Tanaka

**Affiliations:** Department of Obstetrics and Gynecology, The Jikei University School of Medicine, 3-25-8 Nishi-shinbashi, Minato-ku, Tokyo 105-8461, Japan

## Abstract

*Objective*. To investigate the incidence of various antiphospholipid antibodies (aPLs), measured by commercial-based laboratory, with recurrent spontaneous abortion (RSA) patients and the impact of the species, isotype, titer, and number of positive aPLs on reproductive outcome in Japanese. *Method*. In this retrospective cohort study, 263 patients with RSA without possible causes were investigated. Of 131 patients with one or more positive aPL, 82 pregnant women under anticoagulant therapy were evaluated. *Results*. The incidence of various aPLs was almost consistent with previous report. Overall, successful pregnancy rate with anticoagulant therapy was 91.4% regardless of aPL profiles. There was no significant difference in the pregnancy maintenance rate between IgG and IgM groups or single positive and multiple positive groups, but there was a tendency for the rate with aspirin to be lower than with aspirin plus heparin in IgG group. *Conclusion*. aPL profile did not affect the pregnancy maintenance rate when anticoagulant therapy was actively introduced, however in IgG group, we recommend combination therapy with aspirin and heparin.

## 1. Introduction

 Since the pathogenic association of antiphospholipid antibody (aPL) with recurrent spontaneous abortions (RSA) was reported, the efficacy of anticoagulant therapy for patients that have undergone RSA and have positive aPL has been investigated [1–4]. Regarding test items of aPLs for RSA, an international consensus statement concerning the update of the classification criteria for definite antiphospholipid syndrome (APS), published in February 2006 [5], specified only anticardiolipin antibody (aCL) of IgG and/or IgM isotype in a medium or high titer (over the 99th percentile), anticardiolipin-*β*2 glycoprotein-I antibody (a*β*2GPI) of IgG and/or IgM isotype (over the 99th percentile), and lupus anticoagulant (LA). For these patients met with above laboratory criteria and clinical criteria for APS, a treatment combining oral aspirin with the subcutaneous injection of unfractionated heparin has been regarded as superior to aspirin alone [6, 7]. However, various other aPLs, such as antiphosphatidylserine antibody (aPS) [8], antiphosphatidylethanolamine antibody (aPE) [9, 10], and others [11] have been considered to be involved in the etiology of RSA, but these aPLs are not mentioned in these revised classification criteria. Moreover, the relevance of isotypes, titer, or the number of positive aPLs in RSA has not been clarified. In fact, we see many RSA patients, not met the criteria for APS, but had one or more positive aPL. In the meantime, as for the detection of aPLs, laboratory criteria are not always reproducible because of well-known inter- and/or intralaboratory variability [12]. In Japan, a few facilities are testing aPLs in their own laboratory and in many other facilities, aPLs are measured by commercial-based laboratory reporting the 95th percentile and more as positive result, so the interpretation of the result needs to be careful. On the other hand, almost all RSA patients with one or more positive aPL (the 95th percentile and more) prefer actively to be introduced an anticoagulant therapy for their next pregnancy. 

 Given the above background, we examined aPE and aPS of IgG and IgM antibodies in addition to LA, a*β*2GPI, and aCL described in the classification criteria for definite APS and investigated the impact of antibody titers by classifying them into weakly (from the 95th to the 99th percentile) and strongly (over the 99th percentile), antibody isotypes of IgG and IgM, and the numbers of positive antibodies of single and multiple on reproductive outcome. We designed this retrospective cohort study, and our study focused on the incidence of various aPLs with RSA patients, measured by commercial-based laboratory and the impact of their profiles on reproductive outcomes with active anticoagulant therapy in order to develop individualization of treatment for patients with RSA, because the subcutaneous injection of heparin requires daily self-injection during the entire period of pregnancy, so this treatment is very stressful for patients.

## 2. Materials and Methods

### 2.1. Study Design

 This retrospective cohort study was designed to evaluate the incidence of various aPLs, measured by commercial-based laboratory, in patients that had undergone RSA and to determine whether aPL profiles affect reproductive outcomes with the anticoagulant therapy in order to develop individualization of treatment in these patients. The Jikei University Institutional Review Board approved the study design.

### 2.2. Patients

 Records were evaluated for 327 patients that had undergone RSA who visited the outpatient infertility clinic in Jikei University Hospital between November 2005 and July 2009. These patients had suffered 2 or more consecutive spontaneous abortions before the 12th week of gestation (= RSA patients) and were examined by routine checkup for RSA. Then, 64 patients were positive for routine checkup that included anatomical, endocrine, or chromosomal aberration, complications of SLE, or other autoimmune diseases. Of 263 patients negative for routine checkup, various aPLs were measured by commercial-based laboratory, defined 95th percentile and more as positive. Then 132 patients were negative for any aPLs and 131 patients revealed positive for one or more aPL.

 These 131 patients were treated with the anticoagulant therapy, following which, 82 patients conceived. Of the 82 cases of conception, 74 maintained pregnancy successfully, and 8 cases had a miscarriage before the 12th week of pregnancy. Among these miscarriages, 7 cases exhibited normal karyotypes of aborted villi and 1 case had chromosomal aberration. Therefore, with the exception of the case involving chromosomal aberration, a total of 81 cases were analyzed for reproductive outcomes with the anticoagulant therapy. Pregnancy maintenance was defined as the pregnancy continuing beyond the 24th week and resulting in the delivery of a live-born neonate.

**Figure 1:**
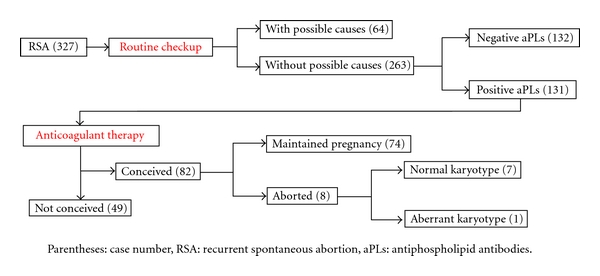
Patients groups and numbers investigated for the incidence of aPLs and reproductive outcomes of the anticoagulant therapy.

Figure 1 shows a summary of the patient groups and numbers investigated the incidences of aPLs and the reproductive outcomes with the anticoagulant therapy.

### 2.3. Measurement of Various aPLs and Definition of the Titer as Positive

 Peripheral blood was drawn from the patients with negative routine checkup for RSA and various aPLs in serum and plasma were measured. At that time, patients were not pregnant. The test items concerning aPLs included LA, a*β*2GPI, aCL-IgG and -IgM, aPE-IgG and -IgM, and aPS-IgG and -IgM. A commercial-based laboratory test company, SRL Laboratory (Tokyo, Japan), measured all these aPLs.

 LA was measured by the dilute Russell's viper venom time method [13] using a kit from Gradipore Ltd. (Australia), and a control clotting time ratio (control individual plasma clotting time/control pooled plasma clotting time) corresponding to the 95th percentile of the value was defined as 1.12, and almost the 99th percentile was 1.3 in normal Japanese population; therefore, the values were divided into two groups, from 1.12 to 1.3 as weakly positive and higher than 1.3 as strongly positive. A a*β*2GPI was measured by ELISA [14, 15] using a kit from Yamasa Co. (Japan), and values from 1.9 (the 95th percentile) to 3.5 (almost the 99th percentile) U/mL were regarded as weakly positive and those higher than 3.5 U/mL as strongly positive. aCL-IgG was measured by ELISA using a MESACUP-IgG kit (MBL Co., Ltd., Japan), and values from 10 U/mL corresponding to the 95th percentile to 20 U/mL (almost the 99th percentile) were regarded as weakly positive and those higher than 20 U/mL as strongly positive. aCL-IgM was measured by ELISA using a kit from SRL Inc. (Japan), and values from 1.0 U/mL corresponding to the 95th percentile to 2.0 U/mL (almost the 99th percentile) were regarded as weakly positive and those higher than 2.0 U/mL as strongly positive. aPE-IgG, aPE-IgM, aPS-IgG, and aPS-IgM were measured as previously described [15, 16], and values from 0.300, 0.450, 1.0, and 1.0 IU/mL, corresponding to the 95th percentile, respectively, calculated from 200 nonpregnant women, to 0.450, 0.750, 1.66, and 1.66 U/mL (almost the 99th percentile), were regarded as weakly positive [16, 17] and those higher than the 99th percentile were regarded as strongly positive. So, in this study, if the test items are 95th percentile and more, the result is defined as positive and anticoagulant therapy was actively introduced.

### 2.4. Patient Treatment Protocols (Anticoagulant Therapy)

 We performed anticoagulant therapy for all patients with one or more aPLs positive RSA women. The anticoagulant therapy consisted of two regimens, aspirin alone or aspirin plus heparin. A daily dose of 100 mg of aspirin was taken orally from prior to conception until the 32nd week of pregnancy, and subcutaneous self-injection of 5,000 units of unfractionated heparin calcium was administered twice a day, starting from the time when the presence of the gestational sac was confirmed in the uterus until the 37th week of pregnancy.

Written informed consent concerning the application of anticoagulant therapies was obtained and the choice between these two regimens was left to patients.

### 2.5. Statistical Analysis

 Differences between the two groups were analyzed for significance with the **χ** ^2^ or Fisher exact test.

## 3. Results

### 3.1. Incidence of Various aPLs, Measured Commercial-Based Laboratory in Patients with RSA

 The incidence of various aPL profiles in two hundreds and sixty-three RSA patients without anatomical, endocrine, or chromosomal aberration, complication of SLE or other autoimmune diseases, was shown in Table 1. Of these 263 patients, the mean age was 33.5 ± 4.7 years, and the mean number of spontaneous abortions was 2.87 ± 1.03. Concerning individual aPL without regarding the titer (weakly or strongly), the positive (the 95th percentile and more) rate of aPS-IgM (73/263: 27.8%) was the highest, followed by aCL-IgM (71/263: 27.0%), LA (47/263: 17.9%), PE-IgG and -IgM (31/263: 11.8%), aCL-IgG (23/263: 8.7%), aPS-IgG (17/263: 6.5%), and finally a*β*2GPI (1/263: 0.4%). As for the incidence of aPLs by isotype, in both aCL and aPS, IgM-isotype was more frequent than IgG-isotype and in aPE, IgM-isotype and IgG-isotype have the same frequency. As for the incidence of aPLs by the titer, in the weakly positive group (from the 95th to the 99th percentile), the positive rates of aPL ranged from 0.4% (1/263: a*β*2GPI) to 19.4% (51/263: aPS-IgM), and in the strongly positive group (over the 99th percentile), those of aPL ranged from 0% (0/263: a*β*2GPI) to 8.4% (22/263: aPS-IgM). Comparing the incidence between these two groups, a higher incidence was found in the weakly positive group in all species of aPLs naturally.

### 3.2. Reproductive Outcomes of the Active Anticoagulant Therapy in RSA Patients with aPL

 The impact of various aPL profiles on reproductive outcomes was shown in Tables 2 and 3. Of the 131 patients treated with the active anticoagulant therapy by the method mentioned above, 82 cases led to conception. After the exclusion of one case with chromosomal aberration of aborted villi, 81 cases were finally analyzed for reproductive outcomes. Total successful pregnancy rate was 91.4% (74/81). For these 81 patients, the mean age was 33.8 ± 4.0 years, and the mean number of spontaneous abortions was 2.8 ± 0.9. When the 81 patients were divided into 74 cases of successfully maintained pregnancy and 7 cases of abortion, the mean ages were 33.8 ± 4.1 and 34.4 ± 4.0 years, and the mean numbers of spontaneous abortions were 2.8 ± 1.0 and 2.7 ± 0.8, respectively. No significant differences were found between these groups. Concerning the variety of aPLs without titer, the rate of pregnancy maintenance of 88.1% (37/42) in aPS-IgM positive cases was the lowest among the various aPLs. However, no significant difference was found between all of each eight aPLs. For instance, the **P** value of the difference between aCL-IgG (the highest rate of pregnancy maintenance: 100%) and aPS-IgM (the lowest rate of pregnancy maintenance: 88.1%) was 0.176, and that of aCL-IgG versus aPS-IgG was 0.277. As for the impact of the titer of aPLs, comparing the rate of pregnancy maintenance in the weakly positive group with that in the strongly positive group for each aPL, no significant difference was found among the six aPLs (IgG and IgM of aCL, aPE, and aPS), and LA or a*β*2GPI strongly positive case was not here. For instance, the lowest **P** value between weakly and strongly positive groups was 0.312 for aPE-IgG. As for the isotype (IgG or IgM group) and the number (single positive or multiple positive group) of positive aPLs, the impact of these on reproductive outcomes was investigated. (Table 3) Eighty-one cases were divided into two groups. The IgM group contained 40 cases with only IgM-positive results and the IgG group contained 41 cases with at least one IgG-positive result. The rate of pregnancy maintenance in the IgM group was 90% (36/40), and that in the IgG group was 92.7% (37/41), showing no significant difference between these two groups (**P** value: 0.667). As for the number of positive aPLs, the rate of pregnancy maintenance in the 34 cases with a single positive group, with only one positive aPL, was 91.2% (31/34) and that in 47 cases with multiple positive group, with two or more positive aPLs, was 91.5% (43/47), also showing no significant difference between these two groups (**P** value: 0.961).

### 3.3. The Therapeutic Outcome of the Two Regimens by the Isotype and Number of Positive aPLs

Finally, we intended to identify a therapeutic principle for RSA patients with aPL.

Table 4 showed how the two regimens of anticoagulant therapy, aspirin alone and aspirin plus heparin, impacted on the reproductive outcomes, showing the results for the isotype of positive aPLs or the number of positive aPLs. There was a tendency for the rate of pregnancy maintenance with aspirin alone to be lower than that with aspirin plus heparin in the IgG group however, this did not constitute a significant difference (*P* value: 0.077). In IgM, single positive and multiple positive group, there was no difference in pregnancy maintenance rate between these two regimens. 

## 4. Discussion

In the field of obstetrics, APS has been mainly investigated with regard to its relationship with pregnancy loss, and therapy for aPL-positive recurrent pregnancy loss has been studied. Although many points concerning the mechanism of aPL-induced pregnancy loss remain unclear, according to a systemic review of RCT by Empson et al. [6], the following results have been shown. (1) A treatment combining low-dose oral aspirin plus twice-a-day subcutaneous injections of unfractionated heparin is beneficial for patients with aPL-positive recurrent pregnancy loss without other causes of infertility, although its efficacy for low-risk patient is not clear. (2) Low-molecular-weight heparin is effective, although whether it exhibits an effect equivalent to that of unfractionated heparin is not clear; the clarification of this issue will require a large-scale RCT. (3) There is no evidence for the efficacy of other therapies such as immunoglobulin and steroid treatments. As a consequence, anticoagulant therapy mainly consisting of a combination of aspirin and unfractionated heparin has become a standard therapy for patients with aPL-positive recurrent pregnancy loss, and currently many facilities perform this treatment as a standard therapy. However, it should be kept in mind that the clinical backgrounds of patients, the aPL species investigated, and the criteria of antibody titers are not necessarily consistent among these reports. The laboratory criteria described in the classification criteria for APS are limited to aCL of IgG or IgM isotype in a medium or higher titer, a*β*2GPI of IgG or IgM isotype, and LA. Sugi et al. reported a higher frequency of aPE in patients with recurrent pregnancy loss before the 10th week of gestation than in healthy women: the frequencies of aPE-IgG, aPE-IgM, and aPE-IgA were 20.1%, 12.2%, and 1.4% in the patients, respectively, which were significantly higher than those in healthy women. These findings suggested that aPE is strongly associated with early pregnancy loss [9] and that aPE testing in addition to aPL examinations of patients with infertility is advisable. Rote et al. detected aPS at higher frequencies (IgG: 87%, IgM: 40%) than aCL (IgG: 68%, IgM: 36%) in patients with idiopathic recurrent pregnancy loss, suggesting a strong association of aPS with early pregnancy loss [8] and indicating the importance of aPS testing. Pregnancy loss was considered to occur at a high frequency in pregnant women with LA and high titers of aCL-IgG [18, 19], and the risks of pregnancy loss and obstetric complications in pregnant women with low titers of aCL-IgG and aCL-IgM were found to be similar to those in pregnant women negative for these antibodies [20], while low titers of aPL were not found to be clinically significant [21]. The clinical significance of the aPL isotypes other than IgG, such as IgM and IgA, including aCL, is still unclear. Matzner et al. investigated 6 species of aPL including aCL in 352 patients with recurrent pregnancy loss and detected aPL in about 60% of the patients, but the antibodies were IgM in 75% of patients [22], suggesting that the IgM antibodies were also pathogenic. However, the rate of pregnancy loss due to aCL-IgM alone was found to be similar to that in aPL-negative women [20]. Moreover, Aoki et al. reported that fetal loss recurred in 27 cases (82%) out of 33 IgG aPL-positive patients when testing aPE, aPS, antiphosphatidylinositol antibody (aPI), and aCL without treatment and in 2 cases (40%) out of 5 IgG aPL-negative but IgM aPL-positive patients, and fetal loss recurred in all 21 patients that were aPL-positive with two or more IgG types. They concluded that the IgG isotype of aPLs was more pathogenic than that of IgM and two or more IgG-positive cases were more at risk than one-IgG-positive cases [23].

In this study, concerning individual aPL without regarding the titer, the positive rates of aCL-IgM and aPS-IgM exceeded 25%, while that of a*β*2GPI was very low, only 0.4%. Those of other aPLs were found to be between 6.5% (aPS-IgG) and 17.9% (LA). This high positive rate, regarded the 95th percentile and more as positive, for aCL-IgM and aPS-IgM in RSA women was five times as high as normal Japanese population, and aCL and aPS may have had stronger association with RSA than a*β*2GPI, consistent with previous reports [8, 22]. With regard to the isotype of aPL, the positive rates of aCL-IgM and aPS-IgM were very high, and those of CL-IgG (8.7%) and aPS-IgG (6.5%) were lower than aCL-IgM and aPS-IgM, suggesting that the IgM isotype of aPLs may also have been pathogenic for early RSA, and these antibodies are worth testing in RSA women. Moreover, strongly positive case (over the 99th percentile) of LA and a*β*2GPI were more scarce than normal Japanese population (0.8% and 0%, resp.), suggesting relatively high association with early RSA and IgG or IgM of aCL, aPE, and aPS. 

With regard to the reproductive outcome of the anticoagulant therapy in patients that had undergone RSA and had any aPLs, the rate of pregnancy maintenance of 88.1% (37/42) in aPS-IgM-positive cases was the lowest, but there was no significant difference among the other eight aPLs in terms of the rate of pregnancy maintenance. There was no LA and a*β*2GPI for strongly positive conceived patient in this study, with regard to the titer of each aPL, upon comparing the pregnancy maintenance rate of weakly positive with strongly positive groups for each aPL, no significant differences were found for all 8 aPLs (Table 2). As mentioned before [18, 19], pregnant women with LA and/or high titer of aCL-IgG were thought to be high risk of RSA, but in fact, the rate of pregnancy maintenance of LA weakly positive RSA patients is 93.8% and that of aCL-IgG strongly positive RSA patients is 100%. No significant difference due to APL species or titer in the pregnancy maintenance rate was identified in this study. Active anticoagulant therapy enabled the normal maintenance of pregnancy regardless of the positive antibody species and titer, and normal pregnancy was maintained in 91.4% of cases on average.

Next, we investigated the impacts of the isotype of aPLs, namely, IgG group or IgM group, and the number of positive aPLs, namely, single or multiple, on reproductive outcomes. All patients with positive LA or a*β*2GPI had other aPLs measured in this study. The rate of pregnancy maintenance in the IgM group was 90.0% and that in the IgG group was 92.7%, showing no significant difference. With regard to the number of positive aPLs, the rate of pregnancy maintenance in 34 cases with single aPL was 91.2% and that in 47 cases with multiple aPLs was 91.5%, also showing no significant difference (Table 3), so we concluded that active anticoagulant therapy for aPL positive RSA patients will result in good pregnancy outcome regardless of the isotype and the number of positive aPLs.

Finally, we intended to identify a therapeutic principle for RSA patients with aPL. There was a tendency for the rate of pregnancy maintenance with aspirin alone to be lower than that with aspirin plus heparin in the IgG group; however, no significant difference was found (**P** value: 0.077), showing a benefit of combination therapy for IgG group and higher pathogenicity in the IgG than IgM group. Moreover, the rate of pregnancy maintenance did not differ between patients treated with aspirin alone and those with a combination of aspirin plus heparin in the IgM group, so aspirin therapy alone is sufficient for the IgM group because heparin therapy was very stressful for patients. As mentioned before [20, 22, 23], the clinical significance of the aPLs isotype of IgM was not clear, but IgM isotype may have had some pathogenicity for early recurrent abortion and may have been weaker than that of IgG.

A limitation of this study may be the consideration of the pregnancy maintenance rate without control patients (without anticoagulant therapy), including patient overlap for aPL positivities and not RCT but retrospective cohort study. This pregnancy maintenance rate is as high as normal Japanese population after an intrauterine pregnancy has been seen by ultrasound, and this study may include normal population because we have resulted from the 95th to the 99th percentile as weakly positive. The interpretation of the result needs to be careful and the accumulation of additional cases and more detailed investigations may be necessary.

## 5. Conclusion

Because IgG or IgM of aCL, aPE, and aPS were more frequent than LAC or a*β*2GPI in RSA patients, we have to test these aPLs for RSA patients checkup. The patients suffering from RSA with some aPLs and do not meet criteria for APS resulted in normal pregnancy outcome by active anticoagulant therapy regardless of the positive antibody species, titer, and the number. In terms of the pathogenicity of aPLs, IgG isotype may be stronger than IgM isotype, so we recommend combination therapy with aspirin and heparin for IgG-positive RSA patients.

## Figures and Tables

**Table 1:** Incidence of various aPL profiles in RSA patients without possible causes.

aPL type and titer	aPL type	aPL titer
	Positive/tested (positive rate; %)	Positive/tested (positive rate; %)
LA		
weakly	47/263 (17.9%)	45/263 (17.1%)
strongly		2/263 (0.8%)

a*β*2GPI		
weakly	1/263 (0.4%)	1/263 (0.4%)
strongly		0/263 (0%)

aCL-IgG		
weakly	23/263 (8.7%)	12/263 (4.6%)
strongly		11/263 (4.2%)

-IgM		
weakly	71/263 (27.0%)	50/263 (19.0%)
strongly		21/263 (8.0%)

aPE-IgG		
weakly	31/263 (11.8%)	22/263 (8.4%)
strongly		9/263 (3.4%)

-IgM		
weakly	31/263 (11.8%)	23/263 (8.7%)
strongly		8/263 (3.0%)

aPS-IgG		
weakly	17/263 (6.5%)	12/263 (4.6%)
strongly		5/263 (1.9%)

-IgM		
weakly	73/263 (27.8%)	51/263 (19.4%)
strongly		22/263 (8.4%)

Positive titers of aPLs were defined as follows:

LA: weakly; from 1.12 to 1.30, strongly; over 1.30.

a*β*2GPI: weakly; from 1.9 to 3.5 U/mL, strongly; over 3.5 U/mL.

aCL-IgG: weakly; from 10 to 20 U/mL, strongly; over 20 U/mL.

aCL-IgM: weakly; from 1.0 to 2.0 U/mL, strongly; over 2.0 U/mL.

aPE-IgG: weakly; from 0.3 to 0.5 U/mL, strongly; over 0.5 U/mL.

aPE-IgM: weakly; from 0.45 to 0.75 U/mL, strongly; over 0.75 U/mL.

aPS-IgG: weakly; from 1.0 to 1.66 U/mL, strongly; over 1.66 U/mL.

aPS-IgM: weakly; from 1.0 to 1.66 U/mL, strongly; over 1.66 U/mL.

**Table 2:** Reproductive outcomes of the anticoagulant therapy according to various aPL profiles in RSA patients.

	Pregnancy					
aPL species and titer	Established^a^ (*n*) total 81	Maintained^a^ (*n*) total 74	Aborted^a^ (*n*) total 7	Success rate (%) 91.4		*P* value (weakly versus strongly)
				Species	Titer	
LA						
weakly	32	30	2	93.8	93.8	—
strongly	0	0	0		—	

a*β*2GPI						
weakly	1	1	0	100	100	—
strongly	0	0	0		—	

aCL-IgG						
weakly	6	6	0	100^∗, ∗∗^	100	—
strongly	8	8	0		100	

-IgM						
weakly	25	24	1	95.0	96.0	0.708
strongly	15	14	1		93.3	

aPE-IgG						
weakly	17	15	2	92.0	88.2	0.312
strongly	8	8	0		100	

-IgM						
weakly	12	11	1	94.1	91.7	0.506
strongly	5	5	0		100	

aPS-IgG						
weakly	6	5	1	90.0**	83.3	0.389
strongly	4	4	0		100	

-IgM						
weakly	28	24	4	88.1*	85.7	0.500
strongly	14	13	1		82.9	

^a^Some patients had multiple positive aPLs.

*P* value for *was 0.176 and for **was 0.227.

**Table 3:** Reproductive outcomes of the anticoagulant therapy according to isotypes and the number of positive aPL in RSA patients.

The isotype or number of positive aPL	pregnancy (number)			Successful pregnancy rate (%)	*P* value
	Conceived	Maintained	Aborted		
IgG group^a^	41	38	3	92.7	a versus b = 0.667
IgM group^b^	40	36	4	90.0	

Single positive group^c^	34	31	3	91.2	c versus d = 0.961
Multiple positive group^d^	47	43	4	91.5	

^a^IgG group.

^b^IgM Group.

^c^Single positive group.

^d^Multiple positive group.

**Table 4:** Reproductive outcomes in patients with RSA with aPL according to the kind of anticoagulant therapy.

kind of therapy	The isotype or number of positive aPL	Pregnancy (*n*)			Successful pregnancy rate (%)	*P* value
		Established	Maintained	Aborted		
Aspirin alone	IgG group^a^	10	8	2	80.0	a versus e = 0.077
	IgM group^b^	16	15	1	93.8	
	Single positive group^c^	15	13	2	86.7	b versus f = 0.519
	Multiple positive group^d^	11	10	1	90.9	

Aspirin + heparin	IgG group^e^	31	30	1	96.8	c versus g = 0.410
	IgM group^f^	24	21	3	87.5	
	Single positive group^g^	19	18	1	94.7	d versus h = 0.937
	Multiple positive group^h^	36	33	3	91.7	

^a^IgG group with aspirin.

^b^IgM Group with aspirin.

^c^Single positive group with aspirin.

^d^Multiple positive group with aspirin.

^e^IgG group with aspirin plus heparin.

^f^IgM Group with aspirin plus heparin.

^g^Single positive group with aspirin plus heparin.

^h^Multiple positive group with combination therapy of aspirin plus heparin.
